# Blood brain barrier breakdown as the starting point of cerebral small vessel disease? - New insights from a rat model

**DOI:** 10.1186/2040-7378-5-4

**Published:** 2013-03-14

**Authors:** Stefanie Schreiber, Celine Zoe Bueche, Cornelia Garz, Holger Braun

**Affiliations:** 1 Department of Neurology, Otto-von-Guericke-University, Leipziger Strasse 44, Magdeburg, 39120, Germany; 2 German Center for Neurodegenerative Diseases (DZNE), Brenneckestrasse 6, Magdeburg, 39118, Germany

**Keywords:** Animal model, Blood brain barrier, Cerebral microbleeds, Cerebral small vessel disease, SHRSP

## Abstract

Cerebral small vessel disease (CSVD, cerebral microangiopathy) leads to dementia and stroke-like symptoms. Lacunes, white matter lesions (WML) and microbleeds are the main pathological correlates depicted in in-vivo imaging diagnostics. Early studies described segmental arterial wall disorganizations of small penetrating cerebral arteries as the most pronounced underlying histopathology of lacunes. Luminal narrowing caused by arteriolosclerosis was supposed to result in hypoperfusion with WML and infarcts.

We have used the model of spontaneously hypertensive stroke-prone rats (SHRSP) for a longitudinal study to elucidate early histological changes in small cerebral vessels. We suggest that endothelial injuries lead to multiple sites with blood brain barrier (BBB) leakage which cause an ongoing damage of the vessel wall and finally resulting in vessel ruptures and microbleeds. These microbleeds together with reactive small vessel occlusions induce overt cystic infarcts of the surrounding parenchyma. Thus, multiple endothelial leakage sites seem to be the starting point of cerebral microangiopathy. The vascular system reacts with an activated coagulatory state to these early endothelial injuries and by this induces the formation of stases, accumulations of erythrocytes, which represent the earliest detectable histological peculiarity of small vessel disease in SHRSP.

In this review we focus on the meaning of the BBB breakdown in CSVD and finally discuss possible consequences for clinicians.

## Introduction

The clinical picture of cerebral small vessel disease (CSVD) is complex since the disease develops insidiously and its etiology is only partly understood. Limited correlations between neurological symptoms, imaging diagnostic and pathological findings impede an early enough diagnostic and therefore therapy. It’s obvious, however, that an illness of rather small blood vessels leads to subtle development of dementia and / or episodes of stroke-like symptoms if neurological sensitive areas are affected by so called strategic infarcts. Lacunes, diffuse white matter lesions (WML, also called leukoaraiosis) and microbleeds are the pathognomonic pathological correlates detectable with modern imaging diagnostic.

Lacunes, found in 6% to 11% of autopsied brains, are defined as scars or remnants of small infarcts with a diameter ranging from 3 to 20 mm predominantly found in the basal ganglia, thalamus, pons, subcortical and cerebellar white matter areas [1-3]. Their development and pathological reasons are poorly understood. Leukoaraiosis, i.e. diffuse WML, is still a category of hyperintensities in magnetic resonance imaging (MRI) diagnostic with no clearly defined histological correlate. Maybe demyelinations could be a potential explanation.

More than 40 years ago Fisher described the “segmental arterial wall disorganization” of small penetrating cerebral arteries defined by vessel enlargement due to wall remodeling with “loss of meshwork”, “hemorraghic extravasation” and deposits of fibrinoid material [1] as one underlying trigger for lacunes. Due to our present knowledge, Fisher’s finding might indicate the small vessel wall fragility with blood brain barrier (BBB) disturbances, a passover of plasma resulting in small vessel wall thickening defined as lipohyalinosis [3,4] and merging into small perivascular bleeds with associated reactive vessel occlusions causing acute infarcts [5].

From the current point of view the chronic small vessel wall alterations found in CSVD comprise three subtypes including lipohyalinosis, arteriolosclerosis and cerebral amyloid angiopathy (CAA) [4]. Especially arteriolosclerosis, characterized by endothelial proliferation, plaque-like accumulations of plasma proteins, lymphocytes and macrophages (microatheroma, [4]), leads to a progressive vessel wall narrowing resulting in a delayed blood supply with hypoperfusion of the adjacent parenchyma possibly indicated by diffuse WML [2,6]. CAA develops as a consequence of amyloid-β(Aβ) protein depositions within basement membranes caused by an age- and hypertension-related failure of Aβ elimination along perivascular lymphatic drainage pathways and is also found in Alzheimer’s disease and mixed dementia [4,6,7].

Moreover, common acute lesions found in CSVD are cerebral microbleeds and primary intracerebral hemorrhages, discussed as consequence of small vessel wall fragility converging with acute hemodynamic changes possibly caused by fluctuations of the blood pressure [2,8]. Microbleeds are well documented and supported by pathological findings.

Occlusions of degenerative altered small vessels, typically found in cerebral macroangiopathy associated with thrombotic or embolic stroke, might be a reasonable explanation for lacunar infarcts, but those occlusions are a rather rare event in brain autopsy of patients with CSVD [3]. Thus, Wardlaw and colleagues supposed in a remarkable review 10 years ago, that a breakdown of the BBB might be, however, a much more frequent reason for cerebral microangiopathy since all its histopathological features seem to be caused, result in or at least being associated with an increased vessel wall fragility [3].

We have recently collected data in the model of spontaneously hypertensive stroke-prone rats (SHRSP) that strikingly support the importance of an early BBB dysfunction for the development of CSVD, which will be discussed in this review.

### SHRSP as animal model for CSVD

Only few animal models have been recognized to be suitable for describing CSVD [9,10]. Although it is unlikely that one animal model reveals all of the histopathological features of CSVD, SHRSP have been identified to be the most suitable one mimicking the degenerative small vessel changes and associated lesions found in human CSVD [9,10].

SHRSP were obtained by selective breeding from spontaneously hypertensive rats (SHR) for the development of spontaneous infarcts [11-13] and exhibit an overactivity of the renin-angiotensin-system [14], a detectability of renin within the vascular walls [15], an altered endothelin system [16] and an increased sympathetic nerve activity [17] potentiated by elevated levels of prostaglandins [18] in their sum leading to an early development of chronic severe arterial hypertension [12].

### Cerebral lesions in SHRSP

SHRSP develop small lacunar-like lesions with a volume ranging from 2 to 57 mm^3^, larger non-territorial infarcts anyhow commonly distributed in specific territories of the large cerebral arteries, rarely covering multiple vascular territories or seen in the border zones of the large cerebral arteries as well as territorial infarcts in some cases affecting the whole hemisphere [5,12,19,20]. This indicates a concurrent vasculopathy of large cerebral arteries and is supported indirectly by degenerative vessel wall changes of large leptomeningeal arteries in SHRSP [21]. However, regarding our own studies of SHRSP, we excluded pathologies of the large cerebral arteries by magnetic resonance angiography (MRA) in those single animals who exhibited an extended territorial infarct [5]. Thus, possibly, at least some of the large infarcts rather develop as a consequence of multiple confluent small infarcts emerged at different time points. This idea is supported by a complex histopathology - including spongy edematous grey matter with micro- and larger cysts (comparable to the “trabeculated cavities” in human lacunes, [1]), pale “cytolytic” neurons, neuronal loss and tissue rarefaction, accumulated astrocytes (comparable to astrogliosis associated with human lacunes, [1]), fibrinoid-eosinophilic depositions and degenerative small vessel wall changes including enlarged perivascular spaces, wall thickening and dilatations - marking the ischemic cerebral lesions in SHRSP and being completely different from those histological changes seen in infarcts resulting from large cerebral artery pathology [19-22].

Ischemic lesions are predominately found in cortical areas (basal ganglia and thalamus are affected secondly commonly; [12,20]) and are associated with local cerebral blood flow reduction shown by MRI and single photon emission compute tomography (SPECT) [5,20]. But also already before overt infarcts are detectable SHRSP exhibit a decline in cortical cerebral blood flow and protein synthesis [23]. In this regard it is important to underline, that Fredriksson et al. nearly 20 years ago already by Evans blue injections demonstrated a long lasting BBB leakage characterized by abundant proteinaceous exudates rich in fibrinogen and occurring near to petechial hemorraghes [21].

In contrast, the exact localization and extent of WML have been rarely investigated in SHRSP and therefore the respective existing data is quite incomplete. Myelin stainings show a rarefication of white matter substance accompanied by a general volume expansion in SHRSP with BBB leakage [21]. For the understanding of the CSVD it would be a worthwhile experimental assignment to address the question whether this histology correlates with the extent of WML in MRI.

### Pathological cascade of CSVD in SHRSP

By genetic factors SHRSP develop a vascular risk profile, which comprises arterial hypertension, insulin resistance and hyperinsulinemia, mixed hyperlipidemia and elevated levels of free fatty acids [24]. This genetic burden is sufficient that SHRSP develop spontaneous ischemic lesions at an age between 8 (male) and 13 to 14 months (female) [5,20]. But what is the beginning of this pathology? What changes in the cerebral small vasculature are detectable first?

**Figure 1:**
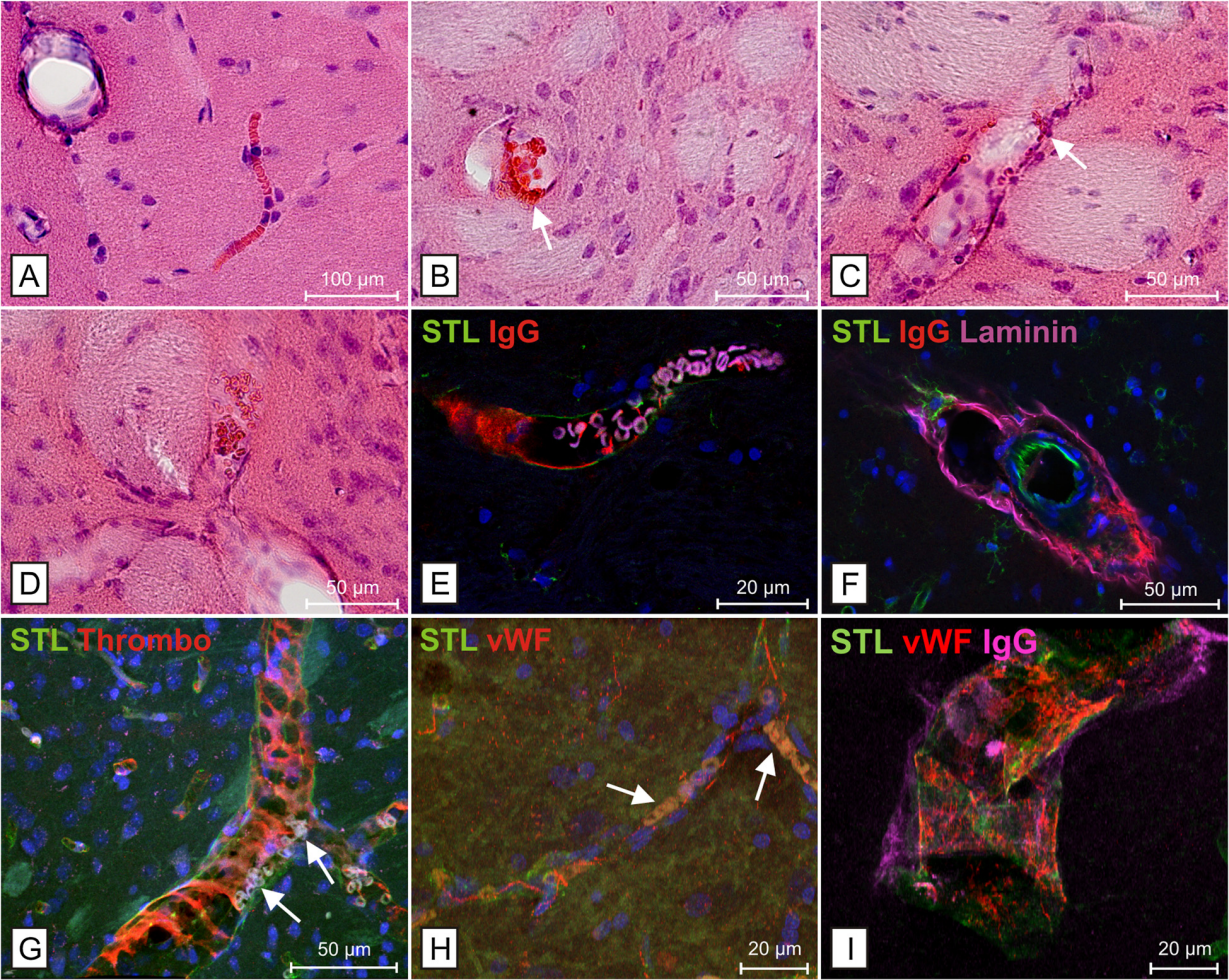
**Stases, BBB disturbances and microvascular dysfunction in SHRSP.** Erythrocyte accumulations (stases) in a capillary (**A**, hippocampus) and arterioles (**B** - **D**, basal ganglia) associated with endothelial injury indicated by beginning diapedesis of erythrocytes (**B** &**C**, white arrows) and IgG-deposits within the wall of affected vessels (**E** &**F**; **E**, erythrocyte autofluorescence in magenta). Stases might be the consequence of erythrocyte accumulations (**G** &**H**, white arrows) within a mesh of thrombocytes (**G**) and threads of the von-Willebrand-factor (vWF; **H**) both activated by blood brain barrier breakdown as illustrated by wall adherence of the vWF in small vessels with IgG deposits (**I**). Note the disturbed vascular tone in small vessels with stases indicated by sausage-like wall dilatations, here shown in a striatal trifurcation of arterioles (**D**). **E** is taken from [25]. **A** – **D** Hematoxylin Eosin (HE) staining. STL - solanum tuberosum lectin used as endothelial marker, Laminin used as basement membrane marker.

Starting a longitudinal study with around 130 SHRSP (animal procedures were conducted after obtaining the approval of the Animal Care Commitee of Sachsen Anhalt; reference numbers of licences for animal testing 42502-2-943, 42502-2-1148, 07/2009 & 08/2012, Magdeburg, Sachsen-Anhalt) we were able to define some kind of pathological cascade which is described in the following: The small vasculature of rather juvenile SHRSP is not distinguishable from age matched Wistar controls. However starting from an age of 12 weeks, in SHRSP we occasionally found in capillaries of the basal ganglia, the hippocampus and cortical areas intraluminal accumulations of erythrocytes (Figure 1A).

**Figure 2:**
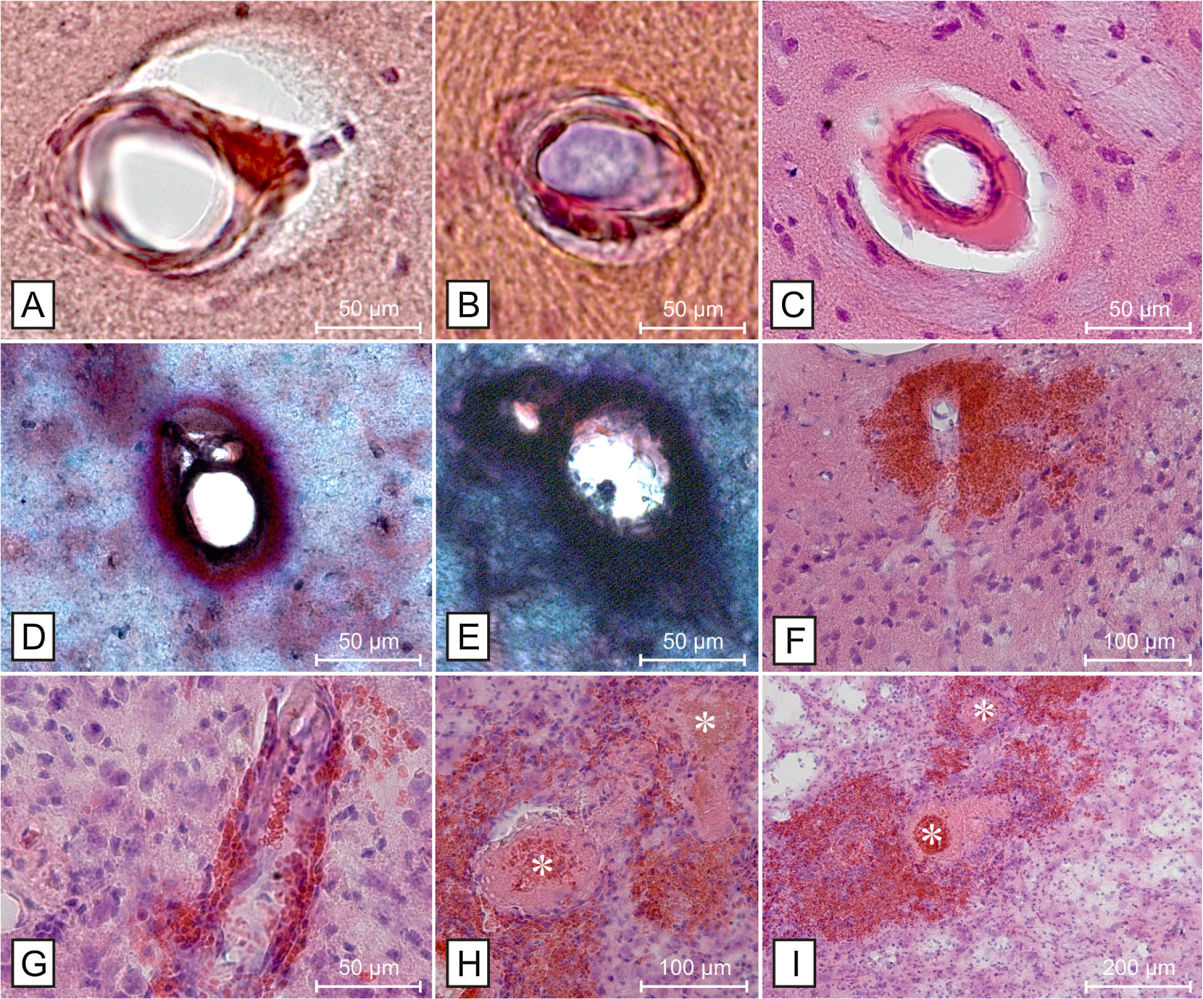
**Degenerative small vessel wall changes, small bleeds and infarcts in SHRSP.** Degenerative small vessel wall changes include arteriolosclerosis (**A** &**B**, hippocampus), lipohyalinosis (**C**, basal ganglia) and thickening of the extracellular matrix (**D**, hippocampus &**E**, basal ganglia) with associated enlarged perivascular spaces (**A** – **C**). Erythrocytes, leaking through injured small vessel walls, form perivascular bleeds (**F** &**G**, cortex). Reactive small vessel occlusions (white asterisks) surrounded by perivascular mini- and microbleeds (**H** &**I**, cortex) with consecutive infarcts consisting of spongy, cystic and hemorrhagic tissue (**I**) are the consequence and represent the final stage of cerebral small vessel disease in SHRSP. **A** &**B** – Congo red staining, **C**, **F** - **I** - HE staining, **D** &**E** – Movat staining.

We refer to this phenomenon as stases indicating an early microvascular dysfunction. With increasing age the number and extent of stases increased significantly; around an age of 28 weeks de facto all SHRSP contained stases in their brain. Now, besides capillaries also arterioles in the basal ganglia, hippocampal and cortical areas, but also, however very rarely, white matter areas including the corpus callosum were affected (Figure 1B-D; [5]). Degenerative small vessel wall changes including arteriolosclerosis, lipohyalinosis and an increase of the extracellular matrix were first detectable at an age of about 18 weeks (Figure 2A-E). None of the SHRSP developed CAA. Around an age of 24 weeks we observed the first animals with mini- and microbleeds ranging in their diameter from 50 to 300 μm and occurred in different brain regions, whereas the basal ganglia and cortical areas were predominantly afflicted (Figure 2F & G; [5]). As an age-dependent phenomenon the number of mini- and microbleeds per animal and the number of affected animals increased up to an age of 44 weeks. Smaller and pronounced infarcts finally occurred at an age of around 30 weeks [5]. Infarcts consist of outstanding spongy areas of necrosis. Noticeable, there was a frequently found concentric arrangement of small hemorrhages caused by leaking erythrocytes around occluded small vessels (Figure 2H & I) leading to a composition of infarcted tissue and bleeds (Figure 2I; [5,19-21]). Occasionally, the site of vessel wall rupture was still identifiable. Therefore we conclude that mini- and microbleeds initiate the phase of infarcts by inducing reactive small vessel occlusions, and, both together cause areas with necrotic damage, representing the final stage of CSVD in SHRSP [5].

### Stases, BBB disturbances and initial microvascular dysfunction in SHRSP

Stases of erythrocytes are the first histological peculiarity of CSVD in SHRSP. The considerable increase of both number and size of the affected small vessels over time is a clear indication, that stases are causally correlated with the pathology of capillaries and arterioles in CSVD. Nevertheless, instantly the question arises: What are the underlying causes of the detected stases?

We could demonstrate, that small vessels affected by intravasal erythrocyte accumulations exhibit deposits of plasmaproteins including immunoglobulin G (IgG) within their walls and within the adjacent perivascular parenchyma indicating associated BBB damage at already young ages (Figure 1E & F; [5,25]).

Another important point is the fact that stases are interwoven with aggregated thrombocytes and multimers of the von-Willebrand factor (vWF) (Figure 1G & H; [25]). This suggests that a local, most likely restricted coagulation is one main reason for their occurrence. A vessel wall adherent activated coagulatory state as a reaction to endothelial injuries (Figure 1I) would therefore comprise a mesh, where erythrocytes aggregate. This would also explain why stases are resistant against transcardial perfusion performed before brain removal and histology. Finally, passive diapedesis of single intravasal erythrocytes from the stases into the perivascular parenchyma provide a further support that endothelial leakages are the main reason for stases (Figure 1B & C; [5]).

Disturbances of autoregulation of small vessel tonus represent another possible reason that at least promotes the formation of stases. This is reflected in constrictions and dilatations of the vessel wall we occasionally found in arteriolar segments with stases (Figure 1D; [5]).

All together stases seem to be an indicator for endothelial injuries inducing a local restricted thrombus formation. Emerging thrombi usually do not lead to a vessel occlusion but remain restricted to the vessel wall site showing a compromised endothelium. Despite the sealing of the leakage by the coagulation system, it can be assumed that certain amounts of plasma still enter the vessel wall [3]. This toxic effect over time makes the vessel wall brashly e.g. simply by cell death of smooth muscle cells or by accumulating plasma proteins between the basal membranes – both described as fibrinoid necrosis and detected as (lipo-)hyalinosis by pathologists [4]. In this regard one has to consider that capillaries are composed only by one endothelial cell layer framed by a basal membrane. Thus leakage of the small vasculature may increase to an amount that locally restricted mini- and microbleeds may occur at a stage when blood vessels still look rather intact. This is actually what we most frequently found in SHRSP. We did not find such a lot of vessels representing the classical lipohyalinosis (Figure 2C). Further our data suggest that mini- and microbleeds are accumulating all over the brain with increasing age (the same as for plasma protein depositions before) and more and more small bleeds develop. This induces reactive small vessel occlusions and necrosis of the surrounding parenchyma.

It is of course unlikely that pathological changes only affect capillaries and arterioles. As already mentioned leptomeningeal arteries also show segmental wall thickening associated with vessel occlusions [21]. Nevertheless, we are convinced that BBB disturbances of small vessels are the main pathologic initiator for the symptoms typical for CSVD. But if so, one could expect that the described vascular pathology is also detectable outside of the brain. Indeed, in SHRSP we were able to demonstrate that accumulations of erythrocytes in peritubular capillaries and glomerular arterioles are also counted among the first histological changes in the kidney. Tubular damage, various stages of glomerulosclerosis and degenerative arteriolar wall changes sometimes combined with occlusions indicate an advanced pathology of hypertensive nephropathy. These findings defined not only the severity of structural renal damage but also were strongly correlated with the CSVD stages in the same rat [26]. We therefore conclude that CSVD is part of a systemic condition, which emerges in the kidney even earlier than in the brain.

### Do SHR exhibit the pathological cascade of CSVD?

Contrary to SHRSP, (stroke-resistant) SHR (SHRSR), an animal model with polygenetic and multifactorial inheritance of chronic arterial hypertension, including renal factors, prostaglandin-adrenergic interactions and abnormalities of the pituitary gland [18], develop a less pronounced blood pressure elevation and exhibit a significant lower incidence of spontaneous tissue “softening” and hemorrhages (about 10% during a life-span of 14 months; [13]). However, after an additional transient occlusion of the middle cerebral artery (MCA) SHR develop stases (“plugging of cerebral microvessels with red blood cells”, [27]), BBB damage, single erythrocyte diapedesis, micro- and macrohemorrhages in the infarcted regions [27-30]. Of course, those MCA infarcts exhibit a territorial pattern, which does not match the common one seen in SHRSP. Stases here might indicate the “no-reflow-phenomenon” of the microvasculature [27,31]. The resulting depletion of oxygen to the endothelium leads to an early BBB leakage, which is exacerbated by an at least partial reperfusion of the capillary bed. A forced diapedesis of blood with consecutive small and larger hemorrhages is the consequence [27,29]. Thus, whereas the pathophysiological link between stases, BBB breakdown and bleeds seems to be comparable between SHRSP and SHR with transient MCA occlusion, the initiation of those histopathological phenomena is completely different from each other. Since the described histopathology is rather and predominantly induced by the large artery occlusion, the described changes are not specific for the model of SHR. Indeed, to our best knowledge, there are so far no investigations of the spontaneous course and the temporal development of microvascular changes in SHR. The sole exception from that is the ken that SHR develop spontaneous disturbances of their BBB indicated by histology, changes in the substantial uptake to and clearance of the cerebrospinal fluid and its protein composition as well as an increased expression of BBB channel proteins [32-37].

Concluding, SHRSP exhibit a complex cascade of CSVD and until now it is unclear, whether SHR develop a comparable natural course of microvascular dysfunction accompanied by histopathological phenomena exceeding their described BBB leakage.

## Conclusions

Thus, coming back to the headline of this review we can state that the SHRSP develops overt infarcts that mainly originate from breakdown of the BBB and not from occluded vessels. Thrombotic occlusions of small penetrating vessels occur only at the end of a pathological cascade originated by endothelial damage. This may have important clinical consequences. Patients with stroke-like symptoms caused by thrombotic or embolic occlusions of large vessels generally benefit from an anti-thrombotic treatment. But the effect of such a treatment could be even detrimental if stroke patients suffer from multiple small vessel lesions originally caused by BBB leakage.

Moreover, one has to ask what is the main difference between SHRSP and controls? Do control rats develop the pathology of CSVD cascade only later on or in other words: Are the vascular risk factors in SHRSP some kind of accelerator for a pathology caused in controls simply by aging? We also found an increasing number of stases in older control rats correlating with vascular changes in the kidney comparable to the findings in SHRSP. Control rats exhibit limited mini- and microbleeds and so far no one exemplar developed overt ischemic lesions. Thus, it is obvious that the vascular system also in control rats undergoes an age-dependent impairment. But probably only a very little fraction of them will develop the typical pathological cascade of CSVD we have found in SHRSP.

The genetically most related population to SHRSP is the SHRSR. The risk profile also includes chronic arterial hypertension with disturbances of renal factors [18]. However, for the development of spontaneous strokes there are additional factors necessary making the difference to SHRSP which are in the end inbred SHR carrying the full risk profile for the pathological cascade leading to stroke.

Thus, besides aging and vascular risk factors we suppose that CSVD pathology needs a certain combination of genetic risk factors and is in its development positively or negatively influenced by lifestyle. In this regard it is very important to mention that the incidence and development of overt infarcts in SHRSP can be heavily promoted by a high salt diet. Moreover, an application of a salt-loaded diet induces an abrupt malign increase of the arterial blood pressure, associated with forced vessel wall ruptures leading to an extended breakdown of the BBB with associated cerebral edema and large primary intracerebral hemorrhages [5,19,20,38]. Thus, high salt uptake not only accelerates the pathological cascade but also dramatically modulates the course of CSVD in SHRSP.

Concluding, the conglomerate of vascular and genetic risk factors in SHRSP obviously favors an initially small and insidious age-dependent developing of endothelial injuries leading to an increasing BBB leakage. The vascular system seems to react with a restricted coagulation which causes stases of erythrocytes as a rather byproduct.

It is a challenge for modern imaging diagnostics to detect these early vascular changes to selectively recognize patients with an elevated vascular risk profile. One approach could be the development of new, especially renal biomarkers in blood and urine. Therefore, in SHRSP we have just started to investigate urine concentrations of the kidney-injury-molecule-1 (KIM-1), marking a renal tubular damage [39]. We are interested whether this marker also indicates the stages of renal and cerebral vascular changes. Modern imaging is surely the second approach for directly visualizing early vascular changes. Contrast-enhanced magnetic resonance microangiography (CE-μMRA; [40]) opens the avenue for new MRI sequences having the capacity to detect tiny vascular events like stases, vessel-wall-adherent coagulation or plasma leakage. A better diagnostic will give the basis for a specifically adapted treatment of patients developing dementia and stroke-like symptoms from CSVD.

Meanwhile our probably best weapon against small cerebral vascular diseases is the promotion of healthy campaigns persuading people to prevent the age driven progress of CSVD under the slogan:

More physical activity, less salt, no smoking!

## Abbreviations

Aβ: Amyloid-β; BBB, Blood brain barrier; CAA: Cerebral amyloid angiopathy; CE-μMRA: Contrast-enhanced magnetic resonance microangiography; CSVD: Cerebral small vessel disease; HE: Hematoxylin Eosin; IgG: Immunoglobulin G; KIM-1: Kidney-injury-molecule-1; MCA: Middle cerebral artery; MRI: Magnetic resonance imaging; MRA: Magnetic resonance angiography; SHR: Spontaneously hypertensive rats; SHRSP: Spontaneously hypertensive stroke-prone rats; SHRSR: Spontaneously hypertensive stroke-resistant rats; SPECT: Single photon emission compute tomography; STL: Solanum tuberosum lectin; vWF: Von-Willebrand factor; WML: White matter lesions.

## Competing interests

The authors declare that they have no competing interests.

## Authors’ contributions

SS, CB, CC and HB wrote the manuscript. All authors read and approved the final manuscript.
